# The NEI Audacious Goals Initiative: Advancing the Frontier of Regenerative Medicine

**DOI:** 10.1167/tvst.10.10.2

**Published:** 2021-08-12

**Authors:** Steven M. Becker, Santa J. Tumminia, Michael F. Chiang

**Affiliations:** 1 Office of Regenerative Medicine, National Eye Institute, Bethesda, Maryland, USA; 2 Office of the Director, National Eye Institute, Bethesda, Maryland, USA

**Keywords:** retina ganglion cell replacement, retina regeneration, photoreceptor replacementIntroduction

## Abstract

Eight years since the launch of the National Eye Institute Audacious Goals Initiative for Regenerative Medicine, real progress has been made in the effort to restore vision by replacing retinal neurons. Although challenges remain, the infrastructure, tools, and preclinical models to support clinical studies in humans are being prepared. Building on the pioneering trials that are replacing the retinal pigment epithelium, it is expected that by the end of this decade first-in-human trials for the replacement of retinal neurons will be initiated.

## Introduction

TVST's special edition on regenerative medicine presents an excellent opportunity for the National Eye Institute to provide an update on its current efforts in this area and summarize the challenges that lie ahead for the field. In 2013, under leadership of then Director, Paul Sieving, the National Eye Institute (NEI) Audacious Goals Initiative (AGI) in Regenerative Medicine set an ambitious goal to “restore vision through the regeneration of neurons and neural connections in the eye and visual system.” This goal builds on the understanding that many leading causes of blindness, like age-related macular degeneration, diabetic retinopathy, and glaucoma, result from degeneration of neuronal cell types in the eye. Attaining the AGI goal is dependent on building the infrastructure necessary to enable the development of strategies to one day replace degenerating or damaged photoreceptors and retinal ganglion cells. Photoreceptors are the cells in the retina that absorb and convert light into electrical signals. Retinal ganglion cells receive visual information from photoreceptors via two intermediate neuron types: bipolar cells and amacrine cells. In most blinding diseases, it is the degeneration of photoreceptors and retinal ganglion cells that lead to vision loss. Aside from prostheses (e.g., Argus II) and gene therapy (e.g., Luxturna) interventions, most current therapies for blinding diseases only hope to slow down the rate of vision loss through neuroprotection of remaining cells.^1^^,^^2^

NEI's regenerative medicine program represents a more nimble approach toward organizing a concerted research effort to reach a clinical translational medicine target within 15 years. This approach includes the AGI being advised by a revolving external oversight group based on programmatic expertise, which functions as a steering committee and assists in planning the AGI's scientific trajectory. This additional oversight allows the initiative to be responsive to rapidly evolving science opportunities directed at the target outcome. It also forces the research enterprise to work backward and deliberately think through the possible steps for clinical translation and directed research rather than the classical approach of casting a wide net with investigator-initiated studies and pursuing the most promising leads into translation. A major challenge to achieving the AGI goal is that neuronal cell replacement therapies in particular have to integrate and reestablish connections to form functional circuits that can restore vision. Current retinal pigment epithelium clinical studies and other studies using bone-marrow derived stem cells do not have to contend with this additional challenge of integration.^3^^–^^5^ The fact that retinal neurons have little turnover throughout life resulted in the pursuit of two distinct strategies for replacement: cell transplantation or the induction of other cell types into retinal neurons. Preclinical cell replacement studies in mice have shown visual function can be restored using embryonic and induced pluripotent stem cell-derived cells, notwithstanding the fact that cytoplasmic material exchange may be a significant factor.^6^^–^^10^ Recent studies have also shown promise in reprogramming cells in vivo to replace lost photoreceptors.^11^^,^^12^

## Building the Infrastructure

NEI has sought to build the knowledge base and technical infrastructure needed to translate these recent preclinical advances to humans. It has focused on catalyzing translation-enabling technology, creating a culture of collaboration, and readily disseminating new information. Since 2015, NEI has launched three research consortia, comprising a total of 16 projects. The first consortium addressed the technical needs and opportunities for imaging cells of the visual system as they respond to light. The second consortium focused on identifying new factors that control cellular regeneration of the visual system. The most recent consortium is developing authentic animal models of human eye disease and using them to test cell replacement strategies. The advances from each of these consortia will provide important contributions to the overall effort.

The functional imaging technologies being developed are needed to enable the evaluation of different cell replacement strategies. NEI has posted these project videos on the NEI AGI webpage.^13^ In addition, a data portal (https://pecan.stjude.cloud/retinalnucleome) hosted by the St. Jude Children's Research Hospital will enable the vision research community to search and explore newly identified regeneration factors. The expectation is that this knowledge will enable improved interventions that target the biological mechanisms needed for cellular regeneration. Lastly, having new animal models should enable the strong preliminary preclinical data needed for Investigational New Drug (IND) applications before human clinical testing.

NEI continues to encourage the research community's efforts to exchange information via its support of scientific meetings and courses. In addition to these traditional dissemination outlets, there is an opportunity to accelerate progress by adopting open science practices and making reagents, cell lines, animal models, and raw data available through deposition into repositories and other exchange platforms to facilitate their widespread uptake and use. Such platforms are listed on the NEI ORM website.

To continue to facilitate progress toward the AGI target goal, the discoveries and technologies that are developed need to be made accessible and disseminated throughout the research community. Communication of these discoveries is key, and the NEI Office of Regenerative Medicine (ORM) is actively promoting research advances through a monthly seminar series highlighting recent publications, hosting symposia to highlight the progress of AGI-funded projects, and offering a regular newsletter that highlights funding opportunities, events, and relevant news. One can sign up to receive updates from the NEI ORM at https://go.usa.gov/xdHBH.

## Remaining Challenges

To inform the AGI program, NEI engaged with the vision community in a variety of ways over the past several years, including requests for information (RFIs), town halls, and workshops. The latest workshop was the AGI Roadmapping Meeting, which occurred in November 2020 and brought together past workshop chairs and key opinion leaders to chart out the next steps for the initiative. NEI has compiled a list of knowledge gaps and barriers to progress that have been summarized in multiple published workshop reports and perspective pieces. A few major challenges that were identified are summarized in Table.

**Table.** Gaps in Knowledge


*Retina Biology*
• Reactivating retina developmental mechanisms
• Inhibition of retinal regeneration by normal aging processes
• Biology of the healthy retinal microenvironment and how disruptions lead to disease
• The plasticity of the retina and consequences of remodeling
*Methods and Tools*
• Label and track transplanted cells longitudinally without fluorescence
• Efficiently reprogram cells to replace lost retinal neurons
• Noninvasively assess restoration of retinal circuitry
*Retinal Transplantation*
• Good manufacturing practices to produce clinical-grade differentiated or precursor cells
• Capacity for integration of cell precursors and factors that promote circuit reformation
• Surgical tools, biomaterials, and scaffolds that may aid to cell delivery
• Managing the immune response

To successfully replace retinal neurons in humans, progress is needed in a number of areas across scientific disciplines. A deeper understanding of homeostasis and how the retina develops and ages, and how different diseases disrupt cell interactions and circuitry is critical to inform how and when interventions should be applied. For exogenous cell replacement, the methods necessary to generate sufficient numbers of replacement cells and the techniques to adequately characterize them for large-scale transplantation trials are still lacking. Alternatively, for endogenous repair approaches, better tools that more effectively reprogram cells into retinal neurons and mobilize them to the sites of damage are needed. There are still technical aspects to be worked out to be able to track and monitor the integration and functional activity of the replaced cells. In addition, after treatment is delivered, proper regulation of the immune response with either immunosuppression or immunomodulatory drugs is required. This necessitates a better understanding of the immune response.

These challenges can all be addressed through multidisciplinary team-based approaches. To support the initiation of early-phase clinical trials to replace retinal neurons to treat eye diseases, NEI is supporting a new clinical trial planning grant program, which will support the development of intervention-based studies.

## Promising Advances in Regenerative Medicine

Multiple advances have been made that have helped the vision community realize the promise of regenerative medicine therapies. Our understanding of how fish and other species regenerate their retinas has been applied so that now neurogenesis can be induced in mouse Müller glia and restore vision.^14^^–^^16^ Endogenous reprogramming strategies are leveraging advances in gene therapy and avoid the manufacturing challenges that face exogenous cell replacement. The discovery and characterization of additional stem cell progenitors in the retina also offers the possibility that other methods to induce and mobilize these niche areas to become active may be harnessed and lead to less-invasive treatments.^17^^,^^18^ Insights into how to promote regeneration of damaged retinal ganglion cell axons have made the idea of replacing RGCs closer to reality as well.^12^^,^^19^^,^^20^

Although additional methods and techniques to support the survival and integration of cell transplants are needed, biomaterials that can aid cell delivery such as hyaluronan–methylcellulose (HAMC) have been developed and scaffolds that could support photoreceptor integration are being optimized and tested.^21^^–^^23^ Perhaps extracellular vesicles, which have shown promise in promoting retinal cell survival and help heal macular holes, can also be coaxed or engineered to promote retinal regeneration in combination with other strategies.^24^^–^^26^ Lastly, elucidation of the roles the different types of glia play in regeneration has made remarkable progress. The role of astrocytes, microglia, and oligodendrocytes in retinal degeneration as well as regeneration has elucidated key mechanisms to reduce inflammatory responses and promote myelination of regenerated axons that will likely need to be incorporated into cell replacement strategies.^27^^–^^30^

Many of the elements needed to realize the promise of restoring vision are coming together, and the clinical knowledge and experience that will result from the current retinal pigment epithelium cell replacement clinical trials will inform the clinical outcome measures, inclusion/exclusion criteria for patients, and study design of future retina neuron cell replacement trials. As immunosuppression regimens and immunomodulatory techniques are tested and refined, there is much optimism that long-term engraftment and integration of retinal neurons will be possible. Eight years since its inception, the NEI AGI has made notable strides and the NEI is committed to marshalling the resources and shepherding the processes necessary to make this ambitious endeavor a reality for patients by the end of the decade.

## References

[1] Luo YH, da Cruz L The Argus II Retinal Prosthesis System. *Prog Retin Eye Res**.* 2016; 50: 89–107, [Pubmed]. 26404104 10.1016/j.preteyeres.2015.09.003

[2] Maguire AM, Simonelli F, Pierce EA, et al. Safety and efficacy of gene transfer for Leber's congenital amaurosis. *N Engl J Med**.* 2008; 358(21): 2240–2248, [Pubmed]. 18441370 10.1056/NEJMoa0802315 PMC2829748

[3] Zarbin M, Sugino I, Townes-Anderson E Concise review: update on retinal pigment epithelium transplantation for age-related macular degeneration. *Stem Cells Transl Med*. 2019; 8(5): 466–477, [Pubmed]. 30748126 10.1002/sctm.18-0282 PMC6477002

[4] Sharma R, Bose D, Maminishkis A, et al. Retinal pigment epithelium replacement therapy for age-related macular degeneration: are we there yet? *Annu Rev Pharmacol Toxicol**.* 2020; 60: 553–572, [Pubmed]. 31914900 10.1146/annurev-pharmtox-010919-023245 PMC8783375

[5] Zakirova EY, Valeeva AN, Aimaletdinov AM, et al. Potential therapeutic application of mesenchymal stem cells in ophthalmology. *Exp Eye Res*. 2019; 189: 107863, [Pubmed]. 31669045 10.1016/j.exer.2019.107863

[6] Mandai M, Fujii M, Hashiguchi T, et al. iPSC-derived retina transplants improve vision in rd1 end-stage retinal-degeneration mice. *Stem Cell Reports*. 2017; 8(1): 69–83, [Pubmed] 28076757 10.1016/j.stemcr.2016.12.008 PMC5233464

[7] Garita-Hernandez M, Lampič M, Chaffiol A, et al. Restoration of visual function by transplantation of optogenetically engineered photoreceptors. *Nat Commun*. 2019; 10(1): 4524, [Pubmed]. 31586094 10.1038/s41467-019-12330-2 PMC6778196

[8] Santos-Ferreira T, Postel K, Stutzki H, et al. Daylight vision repair by cell transplantation. *Stem Cells*. 2015; 33(1): 79–90, [Pubmed]. 25183393 10.1002/stem.1824

[9] Waldron PV, Di Marco F, Kruczek K, et al. Transplanted donor- or stem cell-derived cone photoreceptors can both integrate and undergo material transfer in an environment-dependent manner. *Stem Cell Reports*. 2018; 10(2): 406–421, [Pubmed]. 29307580 10.1016/j.stemcr.2017.12.008 PMC5830910

[10] Nickerson PEB, Ortin-Martinez A, Wallace VA Material exchange in photoreceptor transplantation: updating our understanding of donor/host communication and the future of cell engraftment science. *Front Neural Circuits*. 2018; 12: 17, [Pubmed]. 29559897 10.3389/fncir.2018.00017 PMC5845679

[11] Mahato B, Kaya KD, Fan Y, et al. Pharmacologic fibroblast reprogramming into photoreceptors restores vision. *Nature*. 2020; 581(7806): 83–88, [Pubmed]. 32376950 10.1038/s41586-020-2201-4 PMC7469946

[12] Lu Y, Brommer B, Tian X, et al. Reprogramming to recover youthful epigenetic information and restore vision. *Nature*. 2020; 588(7836): 124–129, [Pubmed]. 33268865 10.1038/s41586-020-2975-4 PMC7752134

[13] National Eye Institute. 2021, Feb 10. AGI Imaging Symposium. https://www.nei.nih.gov/about/goals-and-accomplishments/nei-research-initiatives/audacious-goals-initiative/events-and-reports/agi-imaging-symposium. Accessed February 15, 2021.

[14] Pollak J, Wilken MS, Ueki Y, et al. ASCL1 reprograms mouse Muller glia into neurogenic retinal progenitors. *Development**.* 2013; 140(12): 2619–2631, [Pubmed]. 23637330 10.1242/dev.091355 PMC3666387

[15] Jorstad ML, Wilken MS, Grimes WN, et al. Stimulation of functional neuronal regeneration from Muller glia in adult mice. *Nature**.* 2017; 548(7665): 103–107, [Pubmed]. 28746305 10.1038/nature23283 PMC5991837

[16] Yao K, Qiu S, Wang YV, et al. Restoration of vision after de novo genesis of rod photoreceptors in mammalian retinas. *Nature**.* 2018; 560(7719): 484–488, [Pubmed]. 30111842 10.1038/s41586-018-0425-3 PMC6107416

[17] Saini JS, Temple S, Stern JH Human retinal pigment epithelium stem cell (RPESC). *Adv Exp Med Biol*. 2016; 854: 557–562, [Pubmed]. 26427459 10.1007/978-3-319-17121-0_74

[18] Bernstein SL, Guo Y, Kerr C, et al. The optic nerve lamina region is a neural progenitor cell niche. *Proc Natl Acad Sci USA*. 2020; 117(32): 19287–19298, [Pubmed]. 32723825 10.1073/pnas.2001858117 PMC7431091

[19] Lim JA, Stafford BK, Nguyen PL, et al. Neural activity promotes long-distance, target-specific regeneration of adult retinal axons. *Nat Neurosci*. 2016*;* 19: 1073–1084, [Pubmed]. 27399843 10.1038/nn.4340 PMC5708130

[20] Lina Du Y, Sergeeva EG, Stein DG. Visual recovery following optic nerve crush in male and female wild-type and TRIF-deficient mice. *Restor Neurol Neurosci**.* 2020; 38: 355–368, [Pubmed]. 32986632 10.3233/RNN-201019

[21] Ballios BG, Cooke MJ, van der Kooy D, et al. A hydrogel-based stem cell delivery system to treat retinal degenerative diseases. *Biomaterials**.* 2010*;* 31: 2555–64, [Pubmed]. 20056272 10.1016/j.biomaterials.2009.12.004

[22] Jung YH, Phillips MJ, Lee J, et al. 3D microstructured scaffolds to support photoreceptor polarization and maturation. *Adv Mater**.* 2018; 30(39): e1803550, [Pubmed]. 30109736 10.1002/adma.201803550

[23] Thompson JR, Worthington KS, Green BJ, et al. Two-photon polymerized poly (caprolactone) retinal cell delivery scaffolds and their systemic and retinal biocompatibility. *Acta Biomater*. 2019; 94: 204–218, [Pubmed]. 31055121 10.1016/j.actbio.2019.04.057 PMC6659122

[24] Pan D, Chang X, Xu M, et al. UMSC-derived exosomes promote retinal ganglion cells survival in a rat model of optic nerve crush. *J Chem Neuroanat**.* 2019*;* 96: 134–139, [Pubmed]. 30639447 10.1016/j.jchemneu.2019.01.006

[25] Jordao da Silva-Junior A, Mesentier-Louro LA, Nascimento-Dos-Santos G, et al. Human mesenchymal stem cell therapy promotes retinal ganglion cell survival and target reconnection after optic nerve crush in adult rats. *Stem Cell Res Ther**.* 2021; 12(1): 69, [Pubmed]. 33468246 10.1186/s13287-020-02130-7 PMC7814601

[26] Deng CL, Hu CB, Ling ST, et al. Photoreceptor protection by mesenchymal stem cell transplantation identifies exosomal MiR-21 as a therapeutic for retinal degeneration. *Cell Death Differ**.* 2021; 28(3): 1041–1061. 33082517 10.1038/s41418-020-00636-4 PMC7937676

[27] Todd L, Finkbeiner C, Wong CK, et al. Microglia suppress Ascl1-induced retinal regeneration in mice. *Cell Rep**.* 2020; 33: 108507, [Pubmed]. 33326790 10.1016/j.celrep.2020.108507

[28] Wang J, He X, Meng H, et al. Robust myelination of regenerated axons induced by combined manipulations of GPR17 and microglia. *Neuron**.* 2020*;* 108(5): 876–886, [Pubmed]. 33108748 10.1016/j.neuron.2020.09.016 PMC7736523

[29] Guttenplan KA, Stafford BK, El-Danaf RN, et al. Neurotoxic reactive astrocytes drive neuronal death after retinal injury. *Cell Rep**.* 2020; 31(12): 107776, [Pubmed]. 32579912 10.1016/j.celrep.2020.107776 PMC8091906

[30] Goulart CO, Mendonça HR, Oliveira JT, et al. Repulsive Environment Attenuation during Adult Mouse Optic Nerve Regeneration. *Neural Plast**.* 2018; 2018: 5851914. 30275822 10.1155/2018/5851914 PMC6157103

